# Naturally Occurring Ecdysteroids in *Triticum aestivum* L. and Evaluation of Fenarimol as a Potential Inhibitor of Their Biosynthesis in Plants

**DOI:** 10.3390/ijms22062855

**Published:** 2021-03-11

**Authors:** Anna Janeczko, Jana Oklestkova, Danuše Tarkowská, Barbara Drygaś

**Affiliations:** 1 Polish Academy of Sciences, The Franciszek Górski Institute of Plant Physiology, Niezapominajek 21, 30-239 Kraków, Poland; 2 Laboratory of Growth Regulators, The Czech Academy of Sciences, Institute of Experimental Botany & Palacký University, Šlechtitelů 27, CZ-78371 Olomouc, Czech Republic; danuse.tarkowska@upol.cz; 3 Department of Bioenergetics, Food Analysis and Microbiology, Institute of Food Technology and Nutrition, College of Natural Science, Rzeszow University, Ćwiklińskiej 2D, 35-601 Rzeszow, Poland; badrygas@ur.edu.pl

**Keywords:** cold acclimation, deacclimation, ecdysteroids, fenarimol, plant development, vernalization, winter wheat

## Abstract

Ecdysteroids (ECs) are steroid hormones originally found in the animal kingdom where they function as insect molting hormones. Interestingly, a relatively high number of these substances can also be formed in plant cells. Moreover, ECs have certain regulatory effects on plant physiology, but their role in plants still requires further study. One of the main aims of the present study was to verify a hypothesis that fenarimol, an inhibitor of the biosynthesis of ECs in the animal kingdom, also affects the content of endogenous ECs in plants using winter wheat *Triticum aestivum* L. as a model plant. The levels of endogenous ECs in winter wheat, including the estimation of their changes during a course of different temperature treatments, have been determined using a sensitive analytical method based on UHPLC-MS/MS. Under our experimental conditions, four substances of EC character were detected in the tissue of interest in amounts ranging from less than 1 to over 200 pg·g^−1^ FW: 20-hydroxyecdysone, polypodine B, turkesterone, and isovitexirone. Among them, turkesterone was observed to be the most abundant EC and accumulated mainly in the crowns and leaves of wheat. Importantly, the level of ECs was observed to be dependent on the age of the plants, as well as on growth conditions (especially temperature). Fenarimol, an inhibitor of a cytochrome P450 monooxygenase, was shown to significantly decrease the level of naturally occurring ECs in experimental plants, which may indicate its potential use in studies related to the biosynthesis and physiological function of these substances in plants.

## 1. Introduction

**Figure 1:**
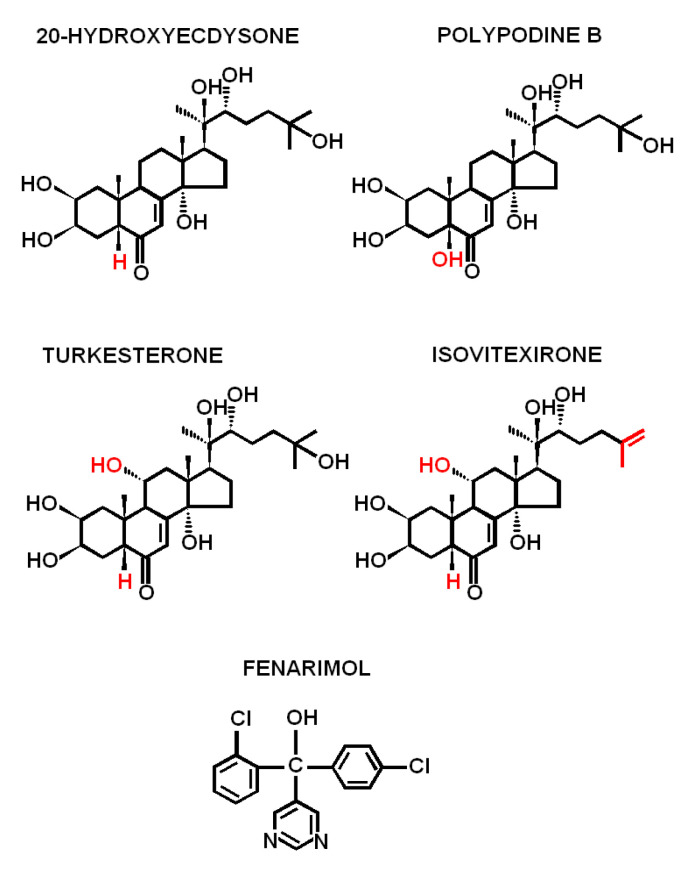
The chemical structure of the selected representatives of ecdysteroids and the structure of fenarimol, the inhibitor of their biosynthesis.

Ecdysteroids (ECs) are mainly known as steroid hormones of arthropods, where they regulate ecdysis and development [1,2,3]. However, ECs are also secondary metabolites widely present in the animal and plant kingdoms [2,4,5,6,7,8]. According to Ecdybase, there are about 400 known ECs, the most common examples being ecdysone or 20-hydroxyecdysone (20E). The group also includes polypodine, turkesterone, isovitexirone or ajugasterone, and many others. ECs are sterol derivatives. Their chemical structure is thus based on a sterane skeleton, and structural differences between particular ECs are connected to the presence of various functional groups in the molecule (Figure 1).

ECs present in plants (also called phytoecdysteroids) are composed of C_19_ and C_29_ -C_30_) atoms [9]. Phytoecdysteroids are most often characterized by a cholest-7-en-6-one carbon skeleton, a 14α-hydroxy-7-en-6-one chromophore, and A/B-cis ring fusion (5β-H) [9]. ECs are of interest to the pharmaceutical industry because they may have multidirectional activity in humans, including antimicrobial, hypoglycemic, hypocholesterolemic, and even anabolic functions [10]. Since chemical synthesis is unprofitable, ECs are obtained directly from the plant material. In vitro cultures may be a valuable solution and source of these compounds [10]. Some taxons are rich in these regulators. For example, more than 50% of the studied species belong to families *Polypodiaceae*, *Pteridaceae*, and *Blechneaceae* [11]. Ecdysteroids are also present in several conifers, and a large number of angiosperm species belong to *Caryophyllaceae*, *Amaranthaceae*, *Chenopodiaceae*, *Asteraceae*, and *Lamiaceae*. In the mentioned plants, ECs can reach concentrations of even 1–2% of the plant’s dry weight [10]. High levels of ECs can be found in species such as *Rhaponticum carthamoides* (Willd.) Iljin., *Rhaponticum nanum* Lipsky, *Rhaponticum integrifolium* C. Winkl., *Serratula inermis* L., and *Serratula algida* Iljin [12]. As for plants that are nutritionally important for humans, spinach (*Spinacia oleracea* L.) and quinoa (*Chenopodium quinoa* Willd.) are also rich in ECs [10]. The taxons that deserve special attention (also from a commercial point of view) may be those used in traditional medicine: *Ajuga turkestanica*, *Leuzea carthamoides* (Willd.) Iljin., and a few *Pfaffia* species in which ECs are particularly abundant in the roots [10,13]. Two monocotyledonous species such as *Cyanotis vaga* (Lour.) Schult. and Schult.f. and *C. arachnoidea* C.B. Clarke, and several species from ferns belonging to *Polypodium*, are also used as sources of ecdysteroid-rich extract [10,14].

In plants, ECs are considered mainly as protection agents (toxins/antifeedants) against harmful insects, but the literature is also rich in data about the effects of exogenously applied ECs on the physiological processes of plants including wheat. Exogenous ECs stimulated the growth of *Triticum vulgare* L., rice, and dwarf-pea internodes [15]. It was found that 20E added to cultures of green algae stimulated cell divisions and major metabolic changes [16] and enhanced the germination of tomato seeds [17]. Pretreatment of wheat with 20E supported germination and growth, despite the presence of heavy metal (Pb) [18]. Foliar spraying with 20E efficiently protected wheat seedlings against salt-oxidative stress [19]. Finally, ECs (mainly 20E) can be positive or negative regulators of photosynthesis, but the phenomenon is dependent on leaf age and is also species-specific [20].

Despite the proven physiological effects of exogenous ECs in plants, there is no certainty that these steroids act as plant hormones. Further experiments on mutants with disturbed biosynthesis/perception of ECs is advisable. Another way forward for studies of the physiological role of ECs in plants would be to use inhibitors of the biosynthesis/perception of ECs. The inhibitor of the biosynthesis of ECs (fenarimol) is known from studies on insects, animals, and generally from medical sciences [21,22,23,24,25,26,27,28]. In agricultural sciences, fenarimol is used as a fungicide [29]. Interestingly, this compound proved to be an inhibitor of the biosynthesis of brassinosteroids (steroid plant hormones structurally related to ECs) [29,30]. However, according to our knowledge, the impact of fenarimol on the biosynthesis of ECs in plants has not been described. As the presence of ECs in wheat, one of the most important cereals in the world, was unknown, these two issues have created the two foundations of the study that is now being presented. Additionally, some preliminary results related to the role of ECs in the development of winter wheat are also a part of this work.

## 2. Results

**Figure 2:**
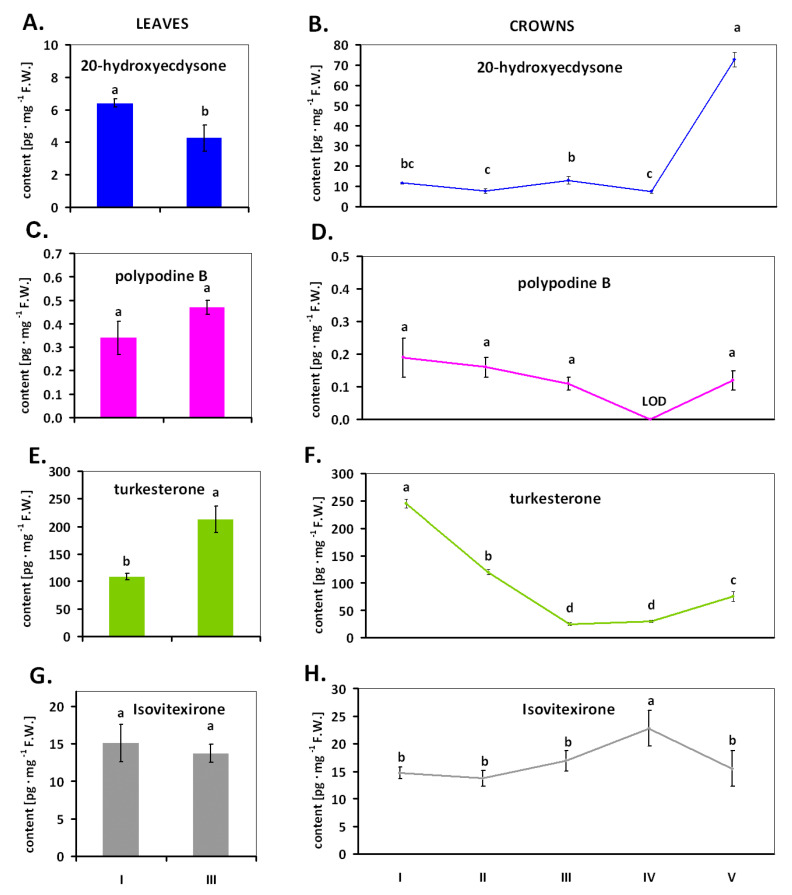
Changes in the content of ecdysteroids in the leaves and crowns of winter wheat. (I) Plants growing 7 days at 20 °C; (II) plants after 7 days at 20 °C and the next 10 days at 5 °C; (III) plants after 7 days at 20 °C and the next 20 days at 5 °C; (IV) plants after 7 days at 20 °C and the next 40 days at 5 °C; (V) plants after 7 days at 20 °C, 40 days at 5 °C and, finally, 10 days at 20 °C. Values marked with the same letters are not significantly different according to Student’s *t*-test (**A**, **C**, **E**, **G**) or Duncan’s test (**B**, **D**, **F**, **H**); *p* ≤ 0.05; LOD—below detection limit.

In winter wheat cv. Grana, four ECs were detected: 20E, polypodine B (polB), turkesterone (tur), and isovitexirone (iso) (Figure 2, experiment 1), with tur being the most abundant substance in this group (Figure 2E,F). It was further observed that the amount of all ECs was dependent on the age, organ (leaves, crowns), and growth conditions (temperature) of the plant.

Notably, 20E was detected in both the leaves and crowns of winter wheat growing at 20 °C (Figure 2A). In leaves, the content of 20E decreased after 20 days of cold (time point III) in comparison with plants grown at 20 °C (time point I). In crowns, the content of 20E was generally higher than in leaves and fluctuated between 7 and 13 pg/mg FW during the entire cold period (Figure 2B). Deaclimation at 20 °C (time point V) caused a 10-fold increase in the content of 20-hydroxyecdysone in the crowns.

The content of polB in leaves was not significantly differentiated and ranged from 0.34 pg·mg^−1^ F.W for plants at 20 °C to 0.47 pg·mg^−1^ FW in plants exposed to cold (Figure 2C). In crowns, the amount of polB ranged from 0.11 to 0.19 pg·mg^−1^ F.W without a statistically significant impact of temperature conditions (Figure 2D). However, its level dropped below the limit of detection of the method used after 40 days of cold exposure (time point IV).

The content of tur in leaves increased significantly in connection with plant exposure to 20 days of cold (from about 100 pg·mg^−1^ FW at a temperature of 20 °C to more than 200 pg·mg^−1^ FW) (Figure 2E). In contrast, in crowns of plants exposed to cold, tur content gradually decreased from 245 pg·mg^−1^ FW (time point I) to about 29 pg·mg^−1^ FW (time point IV). In crowns of plants deacclimated for 10 days by re-exposure to 20 °C, the tur content again increased to 75 pg·mg^−1^ FW (time point V; Figure 2F).

The amount of iso was found at similar levels (about 14–15 pg·mg^−1^ FW) in leaves of plants grown at 20 °C (time point I) and the plants exposed to cold (time point III; Figure 2G). In crowns, notably, the highest content of iso (23 pg·mg^−1^ FW) was detected in the plants subjected to the longest period of cold (time point IV). The iso content in crowns was observed at a similar level in all other time points (I–III and V) and ranged from 14 to 17 pg·mg^−1^ FW. Thus, its levels were similar to those in the leaves.

**Figure 3:**
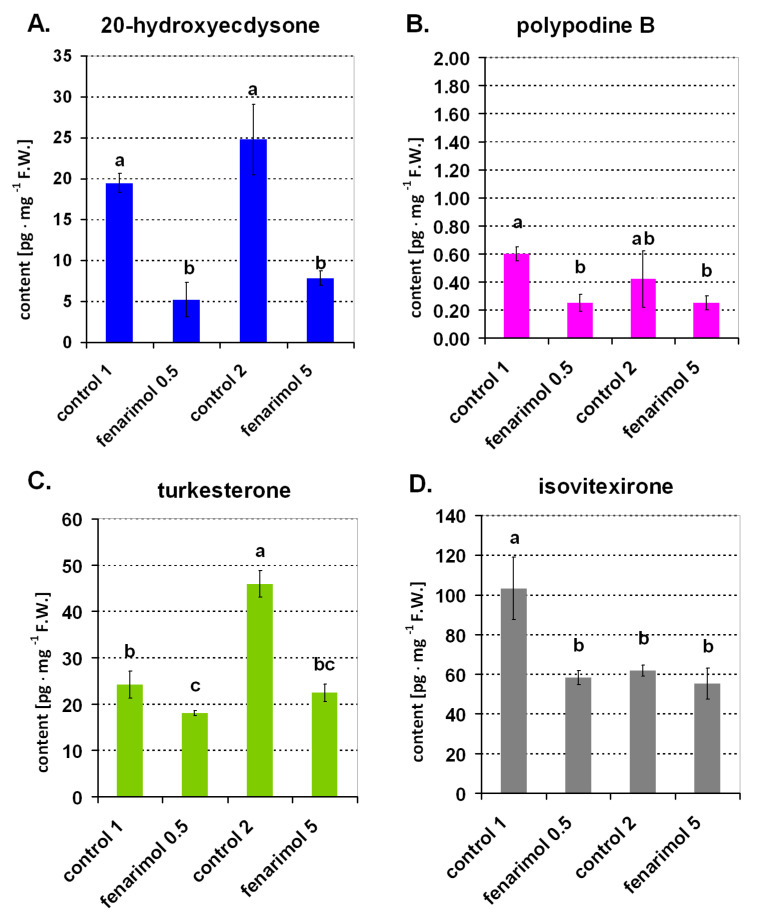
The content of ecdysteroids in the aerial part of 7-d-old seedlings of winter wheat. Plants were grown in Petri dishes in water solutions containing fenarimol at concentrations of 0.5 and 5 mg·dm^−3^ (fenarimol 0.5 and fenarimol 5, respectively). Control 1 is the control for fenarimol 0.5, while Control 2 is the control for fenarimol 5 (for details, see Section 4.2.2). Values marked with the same letters are not significantly different according to Duncan’s test (*p* ≤ 0.05).

As demonstrated in experiment 2, an inhibitor of EC biosynthesis in insect fenarimol also caused a decrease in the content of endogenous ECs in the leaves of experimental plants under our growth conditions (Figure 3A–D). In the aerial part of 7-d-old control wheat seedlings (controls 1 and 2), the level of 20E was detected in the range 19–24 pg·mg^−1^ FW (Figure 3A). The application of fenarimol, at both selected concentrations 0.5 and 5 mg·dm^−3^, caused a reduction in the content of this EC by about 70% (Figure 3A). In the same tissue, the content of the other EC polB reached the level of about 0.5 pg·mg^−1^ FW (Figure 3B), i.e., the application of fenarimol at both concentrations reduced the content of this endogenous EC by about 50% (Figure 3B). The content of tur, which was another EC compound detected in this tissue, was more than 20 pg·mg^−1^ FW in control 1, while in control 2 its content was 46 pg·mg^−1^ FW (Figure 3C). In the first case, application of fenarimol (0.5 mg·dm^−3^) reduced tur content by about 18%, whereas a reduction of over 50% was observed for fenarimol treatment at 5 mg·dm^−3^ (Figure 3C). The iso content in the aerial part of the plants of control 1 was more than 100 pg·mg^−1^ FW, while in the case of control 2 was only about 50 pg·mg^−1^ FW (Figure 3D). The application of fenarimol at 0.5 mg·dm^−3^ led to a reduction of isovitexirone content by more than 40%. The reduction effect was barely visible with the application of 5 mg of fenarimol.

**Figure 4:**
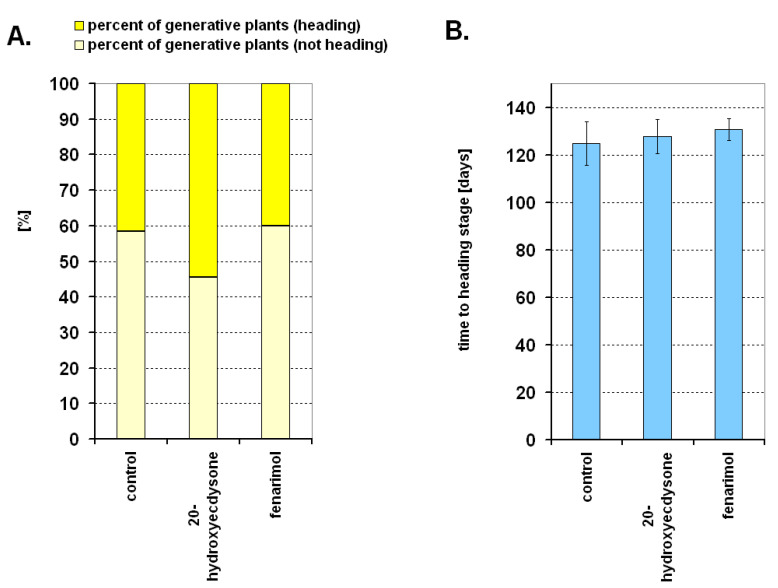
The percentage of winter wheat plants in the generative (not heading and heading) stage of development (**A**) and the time-to-heading stage (days) (**B**) characterizing the control and 20-hydroxyecdysone or fenarimol-treated plants. Statistical analysis was done for (**A**) using test χ^2^, and for (**B**) using Duncan’s test (*p* ≤ 0.05), but no statistically significant changes between treatments were noted.

The results of experiment 3 showed that there was no significant effect of 20E or fenarimol on the generative development of wheat (Figure 4A,B). On average, only about 50% of plants in all treatments (control and treated with regulators) reached the generative heading stage when spikes were visible due to suboptimal vernalization (only 3 weeks of cold instead of 8–9). Time-to-heading was 120–130 days from sowing (Figure 4B). The generative development of the second half of the plants was delayed. Although plants were in the generative stage, spikes were still within the leaf stealth.

## 3. Discussion

Using the sensitive analytical method UHPLC-MS/MS, the presence of ECs was demonstrated in wheat plants for the first time (experiment 1). Four ecdysteroids, 20E, polB, tur, and iso, were detected in the leaves and crowns of wheat. Moreover, ajugasterone C, makisterone C, stachysterone C, and ponasterone A were not found in detectable amounts in the tissues of interest in this study. The presence of 20E, polB, tur, and iso was also confirmed in the aerial part of wheat seedlings cultured in Petri dishes (experiment 2), but their content was slightly modified in comparison to experiment 1. This is probably due to the slightly different sample type (the whole aerial part), different growth conditions, and especially the application of various chemicals (including DMSO not neutral to the plant’s physiology [31]. Generally, the level of ECs in wheat compared to the species rich in these compounds (discussed in more detail in Section 1) was not very high. Interestingly, however, plant steroid hormone brassinosteroid (BRs) was present at a similar level in wheat and barley [32,33,34]. In the case of BRs [32,33,34] and also in the case of ECs, there were changes in the levels of these compounds in wheat depending on factors like the organ examined (leaves, crowns) or growth temperature. This may suggest a physiological role of ECs in wheat. Particularly interesting were the changes in EC levels in leaves and crowns exposed to a certain period of cold. In nature, cold is necessary among other factors to induce metabolic changes leading to the induction of generative development of winter wheat. That is why, in experiment 3, we tried to determine whether (and how) the exogenous EC (20E) affects the generative development of wheat. However, no effect of this regulator was found on the parameters of generative development of winter wheat subjected to suboptimal vernalization. Previously, the same research model established that BRs delay wheat generative development and that another regulator of steroid character (progesterone) accelerates it [35]. In the current experiment, the increase of the ecdysteroid content via exogenous application had a neutral (similar to the control) effect. Additionally, the use of fenarimol (to reduce the EC concentrations during cold exposure) also had no effect on the development of wheat. Thus, changes in the level of ECs occurring under cold conditions do not seem to be unambiguously related to the processes of vernalization and induction of generative development.

On the other hand, it is known from some publications that ECs may stimulate the growth of wheat [15], although according to other studies the exogenous 20E was not active in the growth of wheat coleoptiles [36]. According to our research, the main ECs in wheat tissue seem to be tur rather than 20E (experiment 1, Figure 2E,F), whose levels showed a drastic decrease in wheat crowns during exposure to cold. Assuming that the ECs in wheat may act as growth regulators and that the crowns are connected with the production of tillers, then the observed decrease in the level of turkesterone under cold temperatures could be justified because the growth processes are limited by the cold. Interestingly, increasing the temperature from 5 to 20 °C caused a significant increase in the level of three of the four determined ECs in the crowns as compared to the level recorded in the plants after 40 days of cold exposure. The increase in the level of ECs after the end of the cold period could indicate the role of ECs in growth mechanisms. This is notable considering the fact that, under natural conditions (during spring), elevated temperatures are one of the causes of the resumption of plant growth. On the other hand, it is also well-reported that ECs in plants are necessary for defense against harmful insects due to their ability to cause endocrine disruption and/or the death of phytophagous invertebrates [37,38,39]. This is a common defense mechanism against plant-eating insects and is found in many plants [40]. Thus, an increase in ECs after the end of the cold season could also suggest that the plant somehow prepares itself for pest attacks. Moreover, it is well known from the literature that the content of ECs changes with the plant’s vegetative cycle. In the case of annual plants, ECs are often detectable in newly developed tissues, e.g., young leaves and reproductive organs (like anthers, seeds, and flowers), but less frequently in stems and roots [5]. This may suggest that the highest concentrations of EC are found in tissues that are important for plant survival or organs important for producing the next generation [41]. Furthermore, there is a difference between the distribution of ECs in annual and perennial plants. The annual plants transfer ECs present in seeds to the developing shoots at the beginning of their growth [42], while the perennials often seasonally cycle ECs between their aerial (deciduous) and underground (perennial) parts [43]. However, all these speculations related to the changes in EC content in wheat need to be further verified and supported by experiments to reveal the role of ECs in a plant as economically important as wheat.

In light of this, an important achievement of the work seems to be the confirmation that plants treated with fenarimol showed significantly (up to 70% in the case of 20E) reduced levels of ECs (experiment 2). This opens some possibilities for the use of fenarimol to study the physiological function of ECs in plants. From a practical point of view and due to better effects (the statistically significant reduction in the contents of all four detected ECs), the concentration of fenarimol of 0.5 mg·dm^−3^ is more advisable. The results presented in the paper regarding the level of ECs in plants treated with fenarimol and the control plants were made for samples of 50 mg FW. However, analyses were also performed using larger samples (100 mg, data not shown). Additionally, in this case, a clear decrease in the level of ECs was confirmed under the action of fenarimol. However, the use of larger samples was generally less favorable for the quality of the LC/MS analysis because some ballast and interfering compounds increase background noise. Fenarimol is described in the literature as an inhibitor of a cytochrome P450 monooxygenases (P450s) [23]. Although the biosynthesis ECs in plants is still not described well, it was at least demonstrated that the last step in the biosynthetic pathway of 20E, the main plant EC, is the hydroxylation reaction catalyzed by a cytochrome P450 enzyme [44]. Thus, we can speculate that the mechanism of inhibitory action of fenarimol on the biosynthesis of ECs in wheat plants could be connected to the impact of fenarimol on the P-450 enzyme. Certainly, this requires new experimental data directly supporting this consideration.

Base on the above-described experiments, the main findings can be summarized as follows: (1) in winter wheat, the presence of four naturally occurring ECs, 20-hydroxyecdysone, polypodine B, turkesterone, and isovitexirone, was observed. Their amounts were detected at the level of pg·g^-1^ FW, with turkesterone being the most abundant EC. The levels of these ECs showed a clear dependence on the part of the plant or growth conditions (temperature). (2) Fenarimol, a pyrimidine-type fungicide, had a significant effect on reducing the levels of endogenous ECs in the wheat tissue of interest, which may offer an opportunity to use it as a biosynthetic inhibitor of ECs in further studies dealing with the physiological functions of ECs in plants. (3) There was no effect of exogenous 20-hydroxyedcysone (or fenarimol) on the generative development of wheat.

## 4. Material and Methods

### 4.1. Characteristic of Plant Material

Winter wheat (*Triticum aestivum* L.) cultivar Grana was used in the experiments. The seeds were from the National Centre for Plant Genetic Resources (Radzików, Poland). The cultivar was sourced from Poland but still could be used in scientific experiments as a good model in studies devoted to the thermoperiodical control of generative development (for more information see Janeczko et al. [35]). The cultivar was characterized by the total blockade of generative development, and to break this blockade, the plants required a few weeks (ideally, 8–9) of cold temperatures for the induction of the generative stage (phenomenon called vernalization). Vernalization occurs at cold temperatures similarly to the process of cold acclimation (cold hardening). After 3–6 weeks of plant growth at temperatures 2–5 °C, winter wheat acquires frost tolerance, becoming more tolerant to temperatures below 0 °C. Specific metabolic changes regarding, for example, lipids and sugars, occur in the tissues of plants exposed to cold [45,46]. For plant regrowth both after frost and also during spring, crowns, the meristematic parts localized in the basal section of the plant (near root) and connected to the production of tillers [47,48], are particularly important. That is why, in the current work, crowns are objects of analysis as well as leaves. The hardening effects of cold temperatures can be reversed in some circumstances by exposing winter plants to higher temperatures, i.e., 15 °C (the so-called deacclimation phenomenon). In natural field conditions, deacclimation is particularly dangerous for winter crops in the winter because it lowers their tolerance to frost [49,50].

### 4.2. Experimental Design and Sampling

Three separate experiments were done. The aim of the first experiment was to confirm the presence of ECs in winter wheat plants. Moreover, changes in the content of ECs were also monitored during the growth of winter wheat plants at various temperatures. The plants were grown at 20 °C (control plants not acclimated to cold). Next, the plants were exposed to cold and then re-exposed to 20 °C. The purpose of this was to simulate processes of cold acclimation (and vernalization) and deacclimation (see Section 4.1 and Section 4.2.1). In the second experiment, the effect of fenarimol (the inhibitor of EC biosynthesis usually used in studies on insects) on the level of ECs in wheat seedlings was tested (Section 4.2.2). The aim of the third experiment was to describe the effect of exogenous 20E and fenarimol on the generative development of winter wheat (Section 4.2.3).

#### 4.2.1. Experiment 1. The Presence and Changes of ECs in Winter Wheat

Seeds of wheat were sown in Petri dishes on moisture filter paper and germinated at 24 °C (darkness). The 3-d-old plants were put into pots with soil and cultured in a growth chamber: 7 days at 20 °C (12h photoperiod), next at 5 °C (40 days, 8 h photoperiod), and finally at 20 °C (10 days, 12 h photoperiod). Light intensity was 200 μmol m^−2^ s^−1^ (HPS Philips SON-T AGRO 400 W lamps). Soil mixture: ‘Eco-ziem universal soil’ (Jurków, Poland) from the cultivation plots at the University of Agriculture (Krakow, Poland); sand and ‘Substral Osmocote—a universal substrate’ (Scotts Poland sp. z.o.o., Warsaw, Poland) at a ratio of 4:2:1:2, respectively. Pot size: 40 × 15 × 15 cm; 15 plants per pot. Samples for the analysis of ECs were taken at time-points I–V: (I) plants after 7 days at 20 °C (not acclimated plants); (II) plants after 7 days at 20 °C and next 10 days at 5 °C (10 days of cold acclimation and vernalization); (III) plants after 7 days at 20 °C and next 20 days at 5 °C (20 days of cold acclimation and vernalization); (IV) plants after 7 days at 20 °C and next 40 days at 5 °C (40 days of cold acclimation and vernalization); finally, (V) plants after 7 days at 20 °C, 40 days at 5 °C, and next 10 days at 20 °C (deacclimated plants). Samples of leaves were collected twice (time points I and III). Samples of crowns were collected in all five time-points (I–V). Leaf samples contained 2–3 cm top fragments of the first and second leaves collected from three plants. In the case of crowns, one sample contained crowns from three plants. The number of samples per each time-point was three (*n* = 3). Collected plant material was immediately frozen in liquid nitrogen and stored at −80 °C until being used for purification.

#### 4.2.2. Experiment 2. The Effect of Fenarimol on ECs Content in Wheat

Seeds were germinated on moisture filter paper in Petri dishes (13 cm diameter). After 2 days, germinated plants were moved into new dishes (20 plants per dish). The Petri dishes with plants were divided into four groups, including two controls (control 1 and control 2) and two groups treated with solutions of fenarimol (0.5 and 5 mg·dm^−3^). Fenarimol was purchased from Sigma-Aldrich (Poznan, Poland). The stock solution of fenarimol was made via the dilution of 2 mg of the compound in 0.5 mL of DMSO (Sigma-Aldrich, Poznan, Poland). Aqueous working solutions of fenarimol (0.5 and 5 mg·dm^−3^) were prepared by diluting the stock solution in water. Plants were watered with working solutions of fenarimol on the first day at 50 mL per dish, the second day at 10 mL per dish, and then with distilled water for the next 3 days (30 mL/dish/day). Since both working solutions of fenarimol contained different concentrations of DMSO solvent, two control solutions were prepared containing adequate amounts of DMSO in water. The plants of control 1 were dedicated as controls for plants treated with ‘fenarimol 0.5’, while control 2 was a reference for plants treated with ‘fenarimol 5’. The plants of both controls (1 and 2) were watered with water containing DMSO, 50 mL per dish on the first day, 10 mL per dish on the second day, and then with distilled water for the next 3 days (30 mL/dish/day). During the experiment, the plants were grown in a greenhouse at a temperature of 20 °C day/night under natural solar radiation (November, latitude: 50°30′ North, longitude: 19°55′ East). The aerial parts of the 7-d-old seedlings (usually with only the first leaf and the second one in juvenile form) were collected for analysis of the EC content. One sample contained leaves collected from 2–3 plants. Collected plant material was immediately frozen in liquid nitrogen and stored at −80 °C until being used for purification.

#### 4.2.3. Experiment 3. The Effect of 20-hydroxyecdysone and Fenarimol on Generative Development of Winter Wheat

Wheat seeds were surface-sterilized in a special chamber under a laminar flow of sterile air using a solution containing 96% ethanol, sodium hypochlorite, and HgCl_2_ as described in chapter 2.2.3 of our earlier work [35]. Seed germination took place in in vitro culture in Petri dishes and on basal solid medium (MS) containing macro- and microelements according to Murashige and Skoog [51], with 3% sucrose and 0.6% agar (pH 5.8) [chemicals bought in Sigma-Aldrich (Poznan, Poland)]. On the fourth day, the caryopses were cut off, and isolated embryos with a coleoptile and roots were obtained. Embryos were transferred into Magenta vessels (Sigma-Aldrich, Poznan, Poland; nine embryos per vessel) on MS0 medium (control) and MS media with the addition of 20E (0.5 mg·dm^−3^) or fenarimol (0.5 mg·dm^−3^). Both compounds were bought from Sigma-Aldrich (Poznan, Poland). Stock solutions of 20E and fenarimol were made via the dilution of 1 mg of the compound in 0.5 mL of 96% ethanol. MS media containing 20-hydroxyecdysone or fenarimol were prepared via the dilution of stock solutions into a sterile medium before its solidification (under a flow of sterile air). The proper amount of solvent (ethanol) was also added to the MS0 control medium. Plants transferred on all three media grew in in vitro culture for 4 days at 20 °C + 3 weeks at 5 °C. Temperature/light conditions during the whole culture process were as follows: 4 days of seed germination at 24 °C (12 h photoperiod); isolated embryo growth for 4 days at 20 °C (12 h photoperiod), followed by 3 weeks at 5 °C (suboptimal vernalization; 8 h photoperiod). Light was provided via HPS Philips SON-T AGRO 400 W lamps (Luxmarked, Błonie, Poland). As mentioned in Section 4.1, the tested cultivar Grana requires 8–9 weeks of vernalization for generative induction. A lack of vernalization causes plants to remain vegetative. Suboptimal vernalization (3–6 weeks of cold) causes disturbances in generative development. For example, plant development stops at the early stages of generative differentiation of the apical meristem or the time-of-heading is delayed. Experiments with suboptimal vernalization represent a good model for studying the activity of various compounds (such as 20E and fenarimol here) potentially capable of accelerating or delaying the generative development of winter wheat. In our experiment, after 3 weeks of growth under cold temperatures on media with 20E or fenarimol, plants were transferred from in vitro culture to pots with soil (the soil is described in Section 4.2.1.) and continued growing under greenhouse conditions (20–25 °C, natural light—March/April, latitude: 50°30′ North, longitude: 19°55′ East). Observations of the advancement of wheat development were made every day until the end of the experiment, as described in Section 4.2.2. The number of repetitions was 10 per treatment, where one repetition means one plant.

### 4.3. Measurements and Observations

#### 4.3.1. Extraction and Analysis of Ecdysteroids in Experiments 1 and 2

The analysis of ECs was performed according to the modified protocol of Kamlar et al. [52]. Briefly, the wheat samples were powdered in liquid N_2_ and weighed (50 mg FW), and 1 mL of extraction solvent (80% MeOH, Sigma-Aldrich, Poznań, Poland) was added to each. Ceria stabilized zirconium oxide beads 2 mm in size (Next Advance Inc., Averill Park, NY, USA) were added for further homogenization using a MM 301 vibration mill at a frequency of 27 Hz for 5 min (Retsch GmbH & Co. KG, Haan, Germany). An internal standard ^2^H_6_ labeled ecdysteroid ponasteron A (50 pmol) was added to the samples, and the samples were extracted overnight at 4 °C using a Stuart SB3 benchtop laboratory rotator (Bibby Scientific Ltd., Staffordshire, UK). The homogenates were then centrifuged (36,670 *g*, 15 min, 4 °C; Beckman Avanti™ 30), and the resulting pellets were re-extracted for 60 min using a benchtop rotator at 4 °C and then centrifuged again. The collected supernatants were combined with the first portion from the previous extraction and then were passed through Discovery DPA^®^-6S columns (50 mg, Supelco, Bellefonte, PA, USA), activated with 100% MeOH and equilibrated with 80% MeOH before sample loading. After evaporation to the point of dryness in vacuo (CentriVap^®^, Labconco Corp., MO, USA), the ecdystroid samples were again reconstructed in 50 µL of methanol and analyzed via ultra-performance liquid chromatography with tandem mass spectrometry (UHPLC-MS/MS) an (ACQUITY UPLC^®^ I-Class System, Waters, Milford, MA, USA) with the use of triple-quadrupole mass spectrometer Xevo™ TQ-XS MS (Waters MS Technologies, Manchester, UK).

The chemical structures of ECs shown in Figure 1 were prepared using ChemWindow 3 (ver. 3.0.2, SoftShell, Grand Junction CO, USA) according to data available on http://ecdybase.org/ (accessed on 12 February 2021). The structure of fenarimol in Figure 1 was drawn according to the work of Oh et al. [29].

#### 4.3.2. Evaluation of the Advancement of Wheat Development in Experiment 3

In experiment 3, evaluation of the advancement of wheat development was made based on the estimation of (1) the percent of plants in the generative stage of development—not heading and heading. In the first case (plants not heading), the spike was not visible and remained hidden within the stealth of the leaf. Heading plants are characterized by visible spikes, appearing from leaf stealth. (2) The time to reach the heading stage (in days from the beginning of the experiment). This parameter was estimated only for heading plants. Observations were made for each group separately, including control plants, 20E-treated plants, and fenarimol-treated plants between days 90 and 130 of the experiment. More detailed information regarding this method of evaluation of the advancement of wheat development can be found in chapter 2.3.6 of our earlier work [31].

### 4.4. Statistical Approach

Statistical analysis was performed using software Statistica 6.0 (StatSoft, Tulusa, OK, USA). The figures show mean values; in Figure 2,Figure 3,Figure 4B, mean values are together with the standard deviation (SD). Lowercase letters provided in the figures detail the statistical differences between mean values. Statistical differences were calculated based on Duncan’s test (*p ≤* 0.05)—Figure 2B,D,F,H and Figure 3A–D, or based on Student’s *t*-test—Figure 2A,C,E,G. In the case of Figure 4A, statistical analyses were undertaken using test χ^2^. As for Figure 4B, statistical analyses were undertaken using Duncan’s test (*p ≤* 0.05), but no statistically significant changes were noted. The number of repetitions for the analysis of ECs was three, while for observations of generative development it was 10 (details are given in Section 4.2.1, Section 4.2.2 and Section 4.2.3).
